# Supplementary material for: Responses of Manila Grass (Zoysia matrella) to chilling stress: From transcriptomics to physiology
Source: PLoS One. 2020 Jul 20;15(7):e0235972. doi: 10.1371/journal.pone.0235972 (PMC7371177; doi:10.1371/journal.pone.0235972)
Supplement: Attachment — Submitted filename: Response to Reviewers.docx [file pone.0235972.s015.docx]

To academic editor

1． Please ensure that your manuscript meets PLOS ONE's style requirements, including those for file naming. The PLOS ONE style templates can be found at http://www.plosone.org/attachments/PLOSOne_formatting_sample_main_body.pdf and <http://www.plosone.org/attachments/PLOSOne_formatting_sample_title_authors_affiliations.pdf>

We have downloaded two FDF files: “PLOSOne_formatting_sample_main_body” and “PLOSOne_formatting_sample_title_authors_affiliations”. Our manuscript has been arranged under the guide of the two files.

2. We noticed you have some minor occurrence of overlapping text with the following previous publication(s), which needs to be addressed:

https://www.ncbi.nlm.nih.gov/pubmed/26115186
https://journals.plos.org/plosone/article?id=10.1371/journal.pone.0050785
https://www.tandfonline.com/doi/abs/10.1080/07352689.2014.870411?journalCode=bpts20
https://onlinelibrary.wiley.com/doi/full/10.1111/j.1365-313X.2007.03100.x
<http://www.plantphysiol.org/content/143/2/639.full>

We have revised the manuscript carefully and tried our best to rewrite the overlapping text.

To Reviewer #1

1. Study by Long et al on transcriptomic profiling of Manila grass under cold stress presents alteration in transcripts in response to cold stress and related physiological traits presented as markers of stress. Authors have presented a huge set of information in terms changes in transcript levels involve in various metabolic pathways and physiological process. Although the information presented is useful and can be utilized in related future studies, here in this study the conclusions made by authors are mostly speculative and not interconnected. I agree that 'omics' data is always huge and it is difficult to perform a directional analysis and reach to logical conclusions. A cold stress exposure would certainly change every physiological trait and metabolic pathway in the plant. The necessary part is the analysis of the data, which is entirely missing in this study. Almost everything is covered which has made the things even more complex to understand. Physiological data is very generalized and adds nothing new in the study.
I would highly recommend author to focus the manuscript in a way that can be connected to cold susceptibility or tolerance and exclude rest of the data which is not really serving the purpose. Avoid speculative conclusions as they may be misleading unless validated. Authors are also suggested to reduce the overall length of manuscript with a proper rationale, brief introduction and a more focussed discussion of findings.

Response: Since the transcriptome data is huge and complex. Many clues can be available, but it is not easy to draw firm conclusions, unless corresponding physiological data are also available. In this study, five physiological characters were detected to assist the analysis of the transcriptome data. Among the five physiological parameters, the desecration stress and low bioactive GA level induced by cold are first reported in Manila grass, although they have been reported in other species. Because of the character of transcriptome data, only the direct results were provided in the section of results, most of analysis is arranged in the discussion section. Basing on our transcriptome data as well as the physiology data of our own and of documents, the cold-responding characters in Manila grass were discussed from five aspects: cold sensing and signaling, changes on system and photosynthesis activity, effect on nitrogen metabolism and carbohydrates, metabolism and signaling of plant hormones, and stresses coupled with cold. According to the comment, we read over the manuscript seriously and revised it carefully, especially in the discussion section. In details, in the first aspect, words that interpret the calcium pathway and transcription factors have been reduced; In the second aspect, the activity of mitochondria on photosynthesis has been deleted and the remained content has been reorganized; In the aspect of nitrogen metabolism, words that interpret the transport of nitrate and the assimilation process haves been reduced; in the aspect of plant hormones, only content of GA and CK that could come clear conclusions were remained; In the aspect of multiple stresses, the first paragraph as well the content related to karrikins have been deleted. The revised discussion seems more readable, though there are still many deficiencies need to improve on academic aspect.

To Reviewer #2

1. Do manila grass wilts in the winter or it completes its life cycle?

Response: Manila grass is a type of perennial turfgrass. In winter, the shoots wilts and the stolons are dormant. But if it is freezing severely, the dormant stolons will also die off. The word “perennial” has been supplemented in the introduction as following: For instance, Manila grass (*Zoysia matrella* Merr.), a popular warm season perennial turfgrass, shows excellent tolerance to drought and high temperature stresses, but is sensitive to low temperature. The turf it builds always exhibits a long yellow period in temperate zones or even dies off during winter freezes, which compromises its ornamental value and usage greatly.

2. Plant material and treatment: why this temperature was chosen?

Response: 4℃-cold is often used to treat plant for cold-responding study. So we also used the condition to investigate the cold response in Manila grass.

3．day night hours not mentioned.

Response: The day/night hours are 14h/10h. In the method section, we say: For cold treatment, plants were transferred to a growth chamber set to a 14h-photoperiod.

4．Too long discussion.

Response:The discussion section has been reduced, as described in the response to reviewer #1.

5．Why only leaves not roots were taken for study?

Response: Since leaves are sensitive to low temperature. When the atmospheric temperature drops to 10℃ or lower, leaves turn yellow and wilt gradually.
