# Supplementary material for: Responses of Manila Grass (Zoysia matrella) to chilling stress: From transcriptomics to physiology
Source: PLoS One. 2020 Jul 20;15(7):e0235972. doi: 10.1371/journal.pone.0235972 (PMC7371177; doi:10.1371/journal.pone.0235972)
Supplement: Attachment — Submitted filename: Response to reviewers.docx [file pone.0235972.s016.docx]

Response to reviewers

Reviewer #2: One point is that there is quite difference in cold stress, chilling stress and frost stress. Please check which one fits in your criteria as in grass species all three affects differentially, and change the title accordingly.

Response: Cold stress includes chilling stress (0 - 20 °C) and freezing stress (< 0 °C) (Chinnusamy et al., 2007). Strictly speaking, we should use chilling stress instead of cold stress. Therefore, we has changed the tile to “Responses of Manila Grass (Zoysia matrella) to chilling stress: from transcriptomics to physiology”.

Chinnusamy, V., Zhu, J., & Zhu, J.-K. (2007). *Cold stress regulation of gene expression in plants. Trends in Plant Science, 12(10), 444–451.* doi:10.1016/j.tplants.2007.07.002
